# Machine learning using multimodal and autonomic nervous system parameters predicts clinically apparent stroke-associated pneumonia in a development and testing study

**DOI:** 10.1007/s00415-023-12031-3

**Published:** 2023-10-18

**Authors:** Alexander Nelde, Laura Krumm, Subhi Arafat, Benjamin Hotter, Christian H. Nolte, Jan F. Scheitz, Markus G. Klammer, Michael Krämer, Franziska Scheib, Matthias Endres, Andreas Meisel, Christian Meisel

**Affiliations:** 1 Department of Neurology With Experimental Neurology, Charité—Universitätsmedizin Berlin, Bonhoefferweg 3, 10117 Berlin, Germany; 2 Center for Stroke Research Berlin, Berlin, Germany; 3 Berlin Institute of Health, Berlin, Germany; 4 German Center for Cardiovascular Research (DZHK), Partner Site, Berlin, Germany; 5 NeuroCure Cluster of Excellence, Charité—Universitätsmedizin Berlin, Berlin, Germany; 6 German Center for Neurodegenerative Diseases (DZNE), Partner Site, Berlin, Germany; 7 Bernstein Center for Computational Neuroscience, Berlin, Germany; 8 NeuroCure Clinical Research Center, Berlin, Germany; 9 Einstein Center for Neurosciences, Berlin, Germany

**Keywords:** Stroke associated pneumonia, Machine learning, Autonomic nervous system, Prediction

## Abstract

**Background:**

Stroke-associated pneumonia (SAP) is a preventable determinant for poor outcome after stroke. Machine learning (ML) using large-scale clinical data warehouses may be able to predict SAP and identify patients for targeted interventions. The aim of this study was to develop a prediction model for identifying clinically apparent SAP using automated ML.

**Methods:**

The ML model used clinical and laboratory parameters along with heart rate (HR), heart rate variability (HRV), and blood pressure (BP) values obtained during the first 48 h after stroke unit admission. A logistic regression classifier was developed and internally validated with a nested-cross-validation (nCV) approach. For every shuffle, the model was first trained and validated with a fixed threshold for 0.9 sensitivity, then finally tested on the out-of-sample data and benchmarked against a widely validated clinical score (A2DS2).

**Results:**

We identified 2390 eligible patients admitted to two-stroke units at Charité between October 2020 and June 2023, of whom 1755 had all parameters available. SAP was diagnosed in 96/1755 (5.5%). Circadian profiles in HR, HRV, and BP metrics during the first 48 h after admission exhibited distinct differences between patients with SAP diagnosis vs. those without. CRP, mRS at admission, leukocyte count, high-frequency power in HRV, stroke severity at admission, sex, and diastolic BP were identified as the most informative ML features. We obtained an AUC of 0.91 (CI 0.88–0.95) for the ML model on the out-of-sample data in comparison to an AUC of 0.84 (CI 0.76–0.91) for the previously established A2DS2 score (*p* < 0.001). The ML model provided a sensitivity of 0.87 (CI 0.75–0.97) with a corresponding specificity of 0.82 (CI 0.78–0.85) which outperformed the A2DS2 score for multiple cutoffs.

**Conclusions:**

Automated, data warehouse-based prediction of clinically apparent SAP in the stroke unit setting is feasible, benefits from the inclusion of vital signs, and could be useful for identifying high-risk patients or prophylactic pneumonia management in clinical routine.

## Introduction

Prognosis after stroke is often poor, with more than 40% of patients becoming disabled, institutionalized, or dying within 3 months of the index event [1]. Early medical intervention to treat major modifiable factors may limit mortality and morbidity in stroke [2, 3]. In particular stroke-associated pneumonia (SAP) is consistently associated with a high risk of early mortality in acute stroke [4–6]. The pathogenesis of SAP includes a stroke-induced immunodepression characterized by lymphopenia as well as lymphocytic and monocytic dysfunction impairing antibacterial defenses [14, 15, 30, 32]. The stroke-induced immunodepression may protect against excessive neuroinflammation but increases the risk of post-stroke infections, especially pneumonia [7, 8]. Several clinical parameters are associated with SAP including old age, stroke severity, autonomic dysfunction, impaired consciousness and, most importantly, dysphagia and immune dysfunction [8–10, 33–35]. However, identifying patients at high risk for SAP remains challenging and is currently not broadly implemented in clinical routine despite the availability of widely validated risk scores like the A2DS2 score [15]. The increasing availability of clinical data warehouses for large-scale data acquisition and analysis in many clinical centers may provide novel opportunities for automated, machine learning (ML)-based predictions of SAP and targeted timely interventions.

To gauge SAP risk caused by immune dysfunction, markers related to the intricate interaction between the autonomic nervous system (ANS) and the immune system may be particularly useful. Specifically, while a well-regulated ANS is crucial for maintaining immune homeostasis, stroke may lead to altered cardiovascular function and dysregulation of the ANS, affecting the balance between sympathetic and parasympathetic activity. These changes in the ANS are reflected in the electrocardiography and blood pressure monitoring data of stroke patients [36].

In this retrospective study, we developed, validated and out-of-sample tested a prognostic ML model for predicting the risk of pneumonia after stroke. Taking into consideration ANS and immune system interactions and relying on our previous work [36], in addition to common predictive features based on demographics, comorbidities, and clinical characteristics, we incorporate features derived from electrocardiography and blood pressure monitoring data into an ML model to investigate their impact on SAP prediction for the first time. The model is applicable for automated use in the stroke unit setting during the acute phase after admission.

## Materials and methods

### Dataset

All patients diagnosed with nontraumatic hemorrhagic (ICD-10: I61.-) or ischemic stroke (ICD-10: I63.-) in one of two separate stroke units at Charité—Universitätsmedizin Berlin, Germany, between October 2020 and June 2023 were initially selected. The two stroke units, consisting of a total of 20 monitoring beds, allowed data transfer and integration into the Data Warehouse Connect (DWC) system (Philips) for long-term storage of monitoring data. The Charité/BIH (Berlin Institute of Health) Health Data Lake (HDL), a Hadoop-based platform that allows the storage of a multitude of clinical, epidemiological, laboratory, and monitoring data, was used for further data integration, harmonization, and analysis. Usage and analysis of the data were approved by the Institutional Review Board of Charité—Universitätsmedizin Berlin. Electrocardiogram (ECG) data were recorded with Philips MP30 and MP50 monitors and stored for analysis in the data lake. We collected all beat-to-beat intervals from heartbeats marked by the Philips monitors for up to 48 h after admission. Besides ECG measures, the data lake also contained a comprehensive set of additional parameters from each patient, including blood pressure values, laboratory values, clinical scores, and diagnoses. SAP was diagnosed by the treating physician based on clinical symptoms and/or suggestive clinical examination and/or radiological findings and/or microbiological evidence of pulmonary infection in the stroke unit.

A2DS2—a clinical score to benchmark machine learning (ML)-based prediction.

The A2DS2 score was developed previously as a prognostic score for predicting the risk of pneumonia after ischemic stroke [10]. Table 1 summarizes how the score is composed on an ordinal scale from 0 to 10. We used this score to benchmark ML-based predictions of SAP.

**Table 1:** A2DS2 score

Clinical Variables on Admission	Assigned Points
Age 75 +	+ 1
Atrial fibrillation	+ 1
Dysphagia	+ 2
Sex (male)	+ 1
Stroke severity (NIHSS, National Institutes of Health Stroke Scale)	
0–4	0
5–15 16 +	+ 3 + 5

### Feature selection for machine learning

Our goal was to develop a prediction algorithm for SAP based on data from the first 48 h after stroke unit admission, which would be suitable for automated use in a data warehouse setting. Based on previous studies [10, 11], the following selection of risk factors was coded into features and included in this study for the ML-based prediction of SAP. First, A2DS2 score variables requiring little patient history were collected: age (numeric), sex (binary), and National Institute of Health Stroke Scale (NIHSS, numeric) at admission. Furthermore, we included a modified Rankin Scale (mRS, numeric) at admission and the presence of ischemic or intracranial hemorrhage (I63, binary) as additional features along with laboratory values, including CRP (binary, < 5 mg/l or not) and leukocyte count (binary, within 3.9–10.5 × 10^9^/L or not), that were recorded within 48 h after admission. Finally, based on the available monitoring data, heart rate (HR, from ECG), heart rate variability (HRV, from ECG), and blood pressure (BP) metrics were calculated for the first 48 h after patient admission. Both non-invasive (NBP) and arterial (ABP) blood pressure measurements were included based on availability.

### Calculation of heart rate, heart rate variability, and blood pressure metrics.

HRV measured within 24–72 h after stroke onset has been investigated as a potential prognostic indicator [36–38]. Accordingly, we evaluated HR, HRV, and BP during the initial 48 h after admission.

For the calculation of HR/HRV-associated metrics, we collected heartbeat data in the form of beat-to-beat intervals (RR) of consecutive heartbeats directly from the Philips monitors. Adhering to common standards we divided the data into 5-min segments [39]. Removing ectopic beats entirely from the data led to a high decrease in patient numbers. Therefore, instead of exclusively analyzing normal heartbeat data, we deployed an artifact detection and correction, as well as a detrending method, described by Lipponen and Travainen [41, 42].

**Fig. 1:**
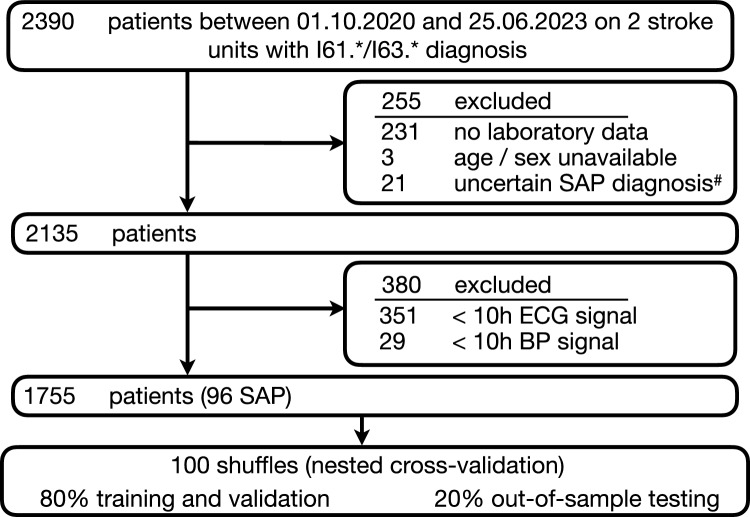
Flow chart of patient selection. # No SAP diagnosis documented in quality management system, but other pneumonia documented within 7 days of admission in a separate data asset; patients thus removed due to unclear classification.

Keeping in mind the circadian variations of the monitoring metrics over the day, we calculated a 24-h time course for each patient and metric. For HR and BP (systolic, diastolic, mean), we derived the median value for each hour of the day from the averaged 5-min segments of the hour, respectively. The median of the HRV metrics was equally obtained by considering the values of all available 5-min segments for the respective hour. Patients were only considered for analysis if all metrics could be derived for at least 10 out of 24 h (Fig. 1).

**Fig. 2:**
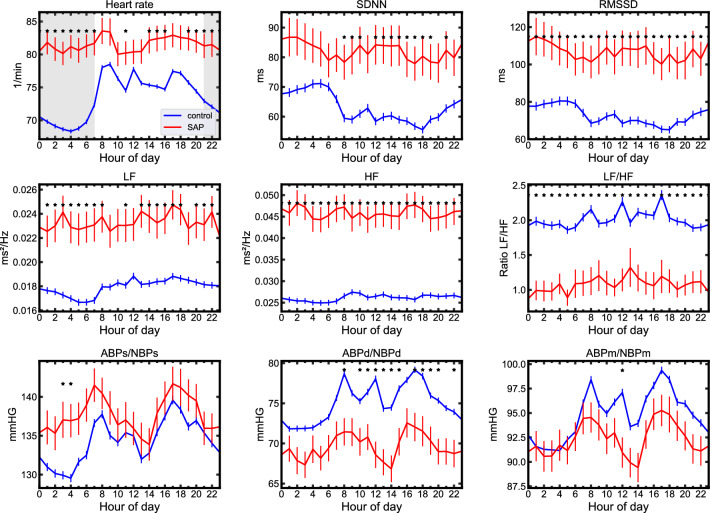
Circadian profiles for the first 48 h after admission in HR, HRV, and BP metrics. Differences between SAP patients (red) and the non-SAP control group (blue) include an overall lower HR and a pronounced dip during the night (gray shaded area), significant differences in all HRV metrics, as well as higher diastolic BP values in the control group. Error bars denote the standard error of the mean. ABP/NBP refers to invasive or non-invasive blood pressure values, whichever were available. *indicates *p* < 0.05 for difference between individual hours; Mann–Whitney-*U*-Test, Bonferroni corrected

Compliant with current field standards, we calculated the following five HRV measures using the NeuroKit2 python toolbox [39, 40]: SDNN (standard deviation of beat-to-beat intervals), RMSSD (root mean square of successive RR intervals), LF (low-frequency power, 0.04–0.15 Hz), HF (high-frequency power, 0.15–0.4 Hz) and the ratio of LF/HF. Finally, ML features were generated by averaging each metric over the 24 values of individual hours, with an exception of the HR, where only values between 21:00 and 7:00 were averaged to better capture the circadian characteristics following previous work [36] (Fig. 2, gray shaded area).

### Machine learning: training, validation, out-of-sample testing

We employed a supervised logistic regression model to predict the development of clinically apparent SAP as a binary classification. We assessed algorithm performance using a nested cross-validation (nCV) approach: The whole data set was split 100 times 4:1 into 80% training and validation sets and 20% out-of-sample testing sets. For reproducibility and comparability, we deployed StratifiedShuffleSplit of the scikit-learn python library with a fixed random state. For every shuffle, we applied a fivefold-CV grid search on the training and validation set, optimizing the area under the receiver-operating curve. Within the grid search, we trained a logistic regression classifier with a newton-cg solver and L2 penalty. Hyperparameters (*C*-values 0.001, 0.1, 1, 10, 100, 1000) were tuned for every fold and the best performing model was selected. Using the selected parameters of the best estimator, the logistic regression classifier was subsequently trained on the entire training and validation set of the respective shuffle. A threshold was then chosen to obtain a sensitivity of 0.9 or larger. This threshold was based on clinical considerations [10, 13]. Finally, these trained models along with their selected thresholds were tested and evaluated on the unseen, out-of-sample test sets and performance metrics were averaged over the 100 shuffles.

The ML pipeline was compiled to balance and normalize the data (using sklearn’s MinMaxScaler). We applied the Synthetic Minority Over-sampling Technique (SMOTE) to balance the two classes (SAP vs. no SAP) during training within the grid search. SMOTE is a form of data augmentation, where new samples are synthesized from existing examples. The new synthetic data points are generated by applying *k*-nearest-neighbors to a random sample of the minority class, then selecting a random member of the resulting *k*-neighbors and finally creating the synthetic sample at a randomly selected point between the initial point and its randomly chosen neighbor in the feature space. This way, synthetic samples are created until the dataset is balanced.

### Performance assessment

We assessed average ML model performance on 100 ensembles of unseen, out-of-sample test sets by calculating the area under the receiver-operator-characteristics (AUC), sensitivity, and specificity for every shuffle. Additionally, we calculated the A2DS2 score [10, 13] and assessed its classifying capabilities on every iteration both on the training and validation set as well as on hold-out test set and benchmarked the results against the ML performance. We obtained 95% confidence intervals of all metrics from bootstrapping (*n* = 200).

## Results

We identified 2390 eligible patients admitted to two stroke units between October 2020 and June 2023 matching our diagnosis criteria (Fig. 1A). From the initial selection, 635 patients were excluded from further analysis due to either (1) missing laboratory or clinical values, (2) because no SAP diagnosis was recorded in the quality management system, but pneumonia was indicated within 7 days after admission in a broader clinical dataset, (3) missing blood pressure data, or (4) insufficient ECG data within the first 48 h (see flowchart in Fig. 1). To fully evaluate the circadian profiles of HR and HRV, we required HR/HRV data to cover at least 10 h in each patient. For the remaining 1755 patients, SAP was diagnosed in 96/1,755 (5.5%). The baseline characteristics of the patients are summarized in Table 2.

**Table 2:** Baseline characteristics

Characteristics	total	SAP	no SAP
No	1,755	96	1,659
Age (years)			
Mean (SD)	73.8 (13.1)	81.1 (8.8)	73.4 (13.1)
Median (IQR)	77.0 (67.0–83.0)	82.0 (75.0–86.2)	77.0 (65.0–83.0)
Male sex, no. (%)	960 (54.7)	46 (47.9)	914 (55.1)
Stroke type, no. (%)			
I61.*	95 (5.4)	10 (10.4)	85 (5.1)
I63.*	1,660 (94.6)	86 (89.6)	1,574 (94.9)
NIHSS (admission)			
Mean (SD)	4.8 (5.6)	12.7 (7.1)	4.4 (5.2)
Median (IQR)	3.0 (1.0–6.0)	12.5 (7.0–18.0)	2.0 (1.0–6.0)
mRS (admission)			
Mean (SD)	2.5 (1.6)	4.3 (1.1)	2.4 (1.6)
Median (IQR)	2.0 (1.0–4.0)	5.0 (4.0–5.0)	2.0 (1.0–4.0)
Dysphagia, no. (%)	44 (2.5)	6 (6.2)	38 (2.3)
Comorbidities, no. (%)			
Hypertension	1360 (77.5)	77 (80.2)	1283 (77.3)
Diabetes mellitus	424 (24.2)	28 (29.2)	396 (23.9)
Atrial fibrillation	453 (25.8)	42 (43.8)	411 (24.8)
Laboratory, mean (SD)			
CRP^#^	0.45 (0.5)	0.96 (0.2)	0.42 (0.5)
Leukocyte count^#^	0.31 (0.46)	0.77 (0.42)	0.29 (0.45)
Monitoring, mean (SD)			
Heart rate (beats/min)^*^	70.9 (13.2)	81.2 (16.6)	70.3 (12.7)
SDNN (ms)	64.5 (53.3)	81.7 (56.9)	63.5 (52.9)
RMSSD (ms)	75.1 (83.3)	105.9 (83.9)	73.3 (82.9)
LF (ms^2^/Hz)	0.0182 (0.0102)	0.0232 (0.0119)	0.0179 (0.0101)
HF (ms^2^/Hz)	0.0272 (0.0259)	0.0456 (0.0281)	0.0262 (0.0254)
LF/HF	2.0 (2.0)	1.1 (1.3)	2.0 (2.0)
Systolic blood pressure (mmHg)	134.3 (16.4)	137.8 (16.4)	134.1 (16.3)
Diastolic blood pressure (mmHg)	74.6 (11.5)	69.7 (13.1)	74.9 (11.3)
Mean blood pressure (mmHg)	94.6 (11.3)	92.4 (11.6)	94.7 (11.2)

^*^Values between 21:00–07:00 h averaged to capture circadian dynamics

^#^Feature coding (binary) explained in Methods section

Figure 2 shows HR, HRV, and BP values for patients with (red) and without (blue) SAP diagnosis. Differences between the groups included an overall lower HR and a pronounced HR dip during the night (gray shaded area), lower values for HRV (with exception for LF/HF), as well as generally higher diastolic BP in the control group. The distinct differences between the groups thus motivated the use of HR, HRV, and BP as additional features in ML. Average values of these metrics were consequently combined with clinical and laboratory features to obtain a feature vector for each patient consisting of age, sex, main diagnosis (I61.- or I63.-), NIHSS at admission, mRS at admission, CRP, leukocyte count, HR, SDNN, RMSSD, LF, HF, LF/HF, systolic BP, diastolic BP and mean BP. These features were selected to allow for maximized automation when implemented in a data warehouse and stroke unit setting, as they only require minimal patient history or clinical tests.

**Fig. 3:**
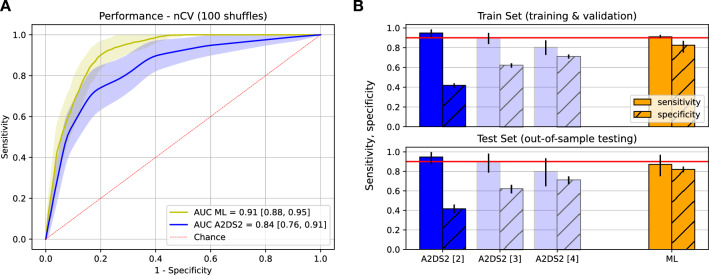
Performance of the trained logistic regression model. **A** averaged receiver-operating-characteristics curves with averaged confidence intervals (filled) for the out-of-sample testing data indicate a performance gain of the ML model in comparison to the A2DS2 benchmark. **B** sensitivity (filled) and specificity (hatched) of the ML model (orange) benchmarked against the A2DS2 score (blue) for different cut-off points (2, 3, 4). With a fixed sensitivity threshold of 0.9 or larger (fixed during validation), the model achieved a higher specificity when compared to the A2DS2 score at similar sensitivity levels in validation and out-of-sample test data. Error bars denote 95% confidence intervals

With these features, we obtained an AUC of 0.91 (95% CI 0.88–0.95) for the ML model on the out-of-sample test data. Similarly, we calculated an AUC of 0.84 (CI 0.76–0.91; Fig. 3A) for the A2DS2 score as a benchmark for our model. ML provided a significantly higher AUC than A2DS2 (*p* < 0.001, Wilcoxon signed-rank test). With the fixed sensitivity thresholds of 0.9 obtained during training and validation, the ML model provided a sensitivity of 0.87 (CI 0.75–0.97) and a corresponding specificity of 0.82 (CI 0.78–0.85) on the out-of-sample test data. The ML model demonstrated superior performance compared to the A2DS2 score, achieving higher specificity at A2DS2 cutoffs of 2 (specificity 0.42, CI 0.37–0.46), 3 (specificity 0.62, CI 0.58–0.66), and 4 (specificity 0.71, CI 0.67–0.75), all while maintaining a high level of sensitivity.

**Fig. 4:**
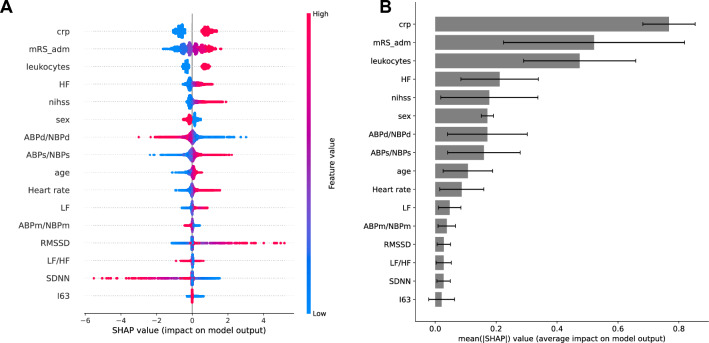
Shapley values (SHAP) indicate feature importance on the model decision for 100 shuffles of nested cross-validation. **A** SHAP summary plot. Positive/negative values indicate the impact of a particular feature to make SAP diagnosis more/less likely, while colors denote whether feature values driving this decision were high or low. **B** SHAP feature importance measured as the mean absolute Shapley values. Error bars indicate standard deviations across 100 shuffles

Shapley values identified the most informative features during training and validation as CRP, mRS at admission, leukocyte count, HF, NIHSS at admission, sex, and diastolic BP followed by systolic BP, age, and HR while LF, LF/HF, SDNN, RMSSD and type of stroke (I63/I61) only had a marginal impact on model classification (Fig. 4).

## Discussion

Our study developed a prognostic ML model for predicting the risk of post-stroke pneumonia during the acute phase of the index event. It is applicable for automated use in the stroke unit. We used clinical and laboratory parameters known to be predictive for SAP, which are routinely collected and do not require extensive history taking or additional tests. We extended these features by including physiological parameters from HR, HRV, and BP obtained during the first 48 h after admission, which exhibited distinct profiles in SAP patients compared to controls.

Importantly, to our knowledge, our study represents the first clinical-scale and ML-based investigation to include heart rate variability (HRV) and associated variables and vitals related to ANS function for SAP prediction. We aimed to integrate these distinct circadian profiles in our model, including the nocturnal non-dipping of heart rate [36]. On out-of-sample data, the ML method provided good discrimination performance between patients developing SAP vs. those that did not, outperforming a previously developed scoring system [10].

Previous research has identified several predictive biomarkers of SAP [15, 26]. Blood-based biomarkers included immune, inflammatory, and stress-related proteins as well as ratios and indices such as the neutrophil-to-lymphocyte ratio (NLR), systemic immune-inflammation index (SII), platelet-to-lymphocyte ratio (PLR), and systemic inflammation response index (SIRI), of which the NLR was reported as the best predictor for SAP occurrence [24]. Heart rate variability [27] and in particular very low-frequency HRV [28], an index of integrative autonomic-humoral control, has been reported as an early marker of sub-acute post-stroke infections, including in experimental models [29]. However, these biomarkers only marginally improved the prediction of SAP over routine clinical parameters [23, 28]. Thus, careful evaluation of prognostic markers is needed [25]. It is reassuring that our data-driven approach identified CRP, leukocyte count, HR, and diastolic BP among the informative ML features which are classical parameters for infection diagnosis.

Using prognostic markers, several SAP prediction scores have been proposed, including the A2DS2 score, the 22-point ISAN score, the PNA score, and the ACDD score. The ICH-LR2S2 score has been developed specifically for SAP after acute intracranial hemorrhage [19]. Comparative internal or external validations of these scores have been performed [13, 20–22]. A large external validation study reported the A2DS2 score to have the highest sensitivity (87%) and the AIS-APS score to have the highest specificity (92.8%) [20]. Another comparative study concluded that the clinical prediction scores varied in their simplicity of use and, while comparable in performance, their utility for preventive intervention trials and in clinical practice required further investigation [21]. More recently, ML-based prediction of SAP, including methods based on natural language processing, has also been explored [17, 18]. These studies reported AUCs of 0.84 which is below the AUC of 0.91 reported here.

In this context, it is important to note that any score and ML model also requires a cutoff or threshold to be provided along with the respective score or ML model to be useful for everyday clinical application and decision support. While AUC is a convenient measure that takes into account many potential cutoffs or thresholds to quantify the general discriminative power of a score or ML model, a pre-determined threshold is required to be established for clinical use. Many studies have not provided such a pre-determined, fixed cutoff based on which to derive sensitivity and specificity when externally testing the performance. By choosing the best threshold post hoc, sensitivity, and specificity values may thus potentially appear over-optimistic. In contrast, we here determined a threshold from training data only and based on clinical considerations (i.e., sensitivity equal or above 0.9) to make the approach applicable for real-world use. We then applied this fixed threshold to the out-of-sample test data for evaluation of sensitivity and specificity.

The development of a prediction score that identifies stroke patients at risk for stroke-associated pneumonia has important clinical implications. By identifying high-risk patients, healthcare providers could take proactive steps to prevent the development of pneumonia, like to intensify methods of pneumonia prophylaxis such as implementing measures to reduce the risk of aspiration (such as optimizing the patient's position during feeding and adapting food consistency), targeted speech and language therapy, more in-depth clinical examinations as well as more frequent blood tests to check for signs of infection [12, 31].

While preventive antibiotic therapy did not improve functional outcomes after stroke, local immunomodulation could open up a new research opportunity to find preventive management for SAP [15, 26]. The benefits of robust SAP prediction regarding patient wellbeing, but also health care costs could be substantial: shorter hospital stays, less—or timelier and more targeted—use of expensive antibiotics with potential side effects (which could slow the development of antibiotic resistances), and better long-term outcome after stroke and much more. In this context, it is an interesting question whether the altered HRV biomarkers analyzed here could also serve as potential targets for preventive measures. Exploring the therapeutic implications and the possibility of mitigating the risk of aspiration pneumonia by modulating HRV should be a relevant focus of future research.

Limitations of this study are inherited in its retrospective setting. Prospective validation in an external patient cohort would enhance validity. In our study, the presence of SAP was defined according to the discretion of the treating physician. Although the diagnosis of SAP follows PISCES recommendations, standardized recording and evaluation of all diagnostic criteria in each case would improve interpretation of the results [16]. Compared with previous internal and external validation studies, we found a surprisingly high level of discrimination for the A2DS2 score. Previous analyses showed this capability to be highly dependent on the thoroughness of the SAP definition applied. However, the reported frequency of SAP in this cohort is well in line with previous studies. The A2DS2 score exhibited similar performance compared to previous studies [10]. From the ML perspective, the comparatively low frequency of SAP in both the training and the testing datasets is a challenge, as in unbalanced datasets ML algorithms tend to classify all instances as the majority class (in this case, no SAP), if not addressed properly. We have tried to solve this problem by using the widely used Synthetic Minority Over-sampling Technique (SMOTE) which helped to improve the performance of our algorithm. Finally, it will be interesting to replicate our results in other post-stroke infections, such as urinary tract infections (UTIs) or colitis. We here chose to focus on SAP as the target outcome as it is the most common post-stroke infection that occurs only a few days after stroke. Future work should determine the generalizability of our approach to other post-stroke infections.

In summary, our results show that automated, data warehouse-based predictions of clinically apparent SAP in the stroke unit setting are feasible and benefit from including parameters of autonomic nervous system function. Such predictions could be useful for identifying high-risk patients, tailor monitoring, and facilitate studies on prophylactic pneumonia management in clinical routine. Future prospective validation studies, however, are needed to fully assess its performance and generalizability prior to a potential implementation into the clinical routine.

## Data Availability

The datasets generated and analyzed during the current study are not publicly available due to privacy or ethical restrictions but may be available from the corresponding author on reasonable request. Access to the data will be granted to qualified researchers for purposes of reproducing the results or replicating the procedure.
